# Incidence of Ganciclovir Resistance in CMV-positive Renal Transplant Recipients and its Association with UL97 Gene Mutations

**Published:** 2017

**Authors:** Hamid Reza Aslani, Shadi Ziaie, Jamshid Salamzadeh, Sara Zaheri, Fariba Samadian, Shayan Mastoor-Tehrani

**Affiliations:** a *Department of Clinical Pharmacy, School of Pharmacy, Shahid Beheshti University of Medical Sciences, Tehran, Iran. *; b *Department of Nephrology and Kidney Transplantation, , Shahid Labbafinejad Medical Center, Shahid Beheshti University of Medical Sciences, Tehran, Iran. *; c *Department of Pathology, School of medicine, Jundishapour University of Medical Sciences, Ahwaz, Iran*; d *School of Pharmacy, Shahid Beheshti University of Medical Sciences, Tehran, Iran.*

**Keywords:** Cytomegalovirus, ganciclovir, UL97, Transplant Recipients, Resistance, Mutations

## Abstract

Human cytomegalovirus (CMV) remains the most common infection affecting organ transplant recipients. Despite advances in the prophylaxis and acute treatment of CMV, it remains an important pathogen affecting the short- and long-term clinical outcome of solid organ transplant recipient. The emergence of CMV resistance in a patient reduces the clinical efficacy of antiviral therapy, complicates therapeutic and clinical management decisions, and in some cases results in loss of the allograft and/or death of the patient. Common mechanisms of CMV resistance to ganciclovir have been described chiefly with the UL97 mutations. Here we evaluate Incidence of ganciclovir resistance in 144 CMV-positive renal transplant recipients and its association with UL97 gene mutations. Active CMV infection was monitored by viral DNA quantification in whole blood, and CMV resistance was assessed by UL97 gene sequencing. Six mutations in six patients were detected. Three patients (2.6%) of 112 patients with history of ganciclovir (GCV) treatment had clinical resistance with single UL97 mutations at loci known to be related to resistance (including mutations at codon 594, codon 460, and codon 520). three patients who were anti-CMV drug naïve had single UL97 mutations (D605E) without clinical resistance. Our results confirm and extend our earlier findings on the specific mutations in the UL97 phosphotransferase gene in loci that have established role in ganciclovir resistance and also indicate that clinical ganciclovir resistance due to UL97 gene mutations is an issue in subjects with history of with ganciclovir treatment. D605E mutations remains a controversial issue that needs further investigations.

## Introduction

Human cytomegalovirus (CMV) is a member of the Betaherpesviridae subfamily that belongs to Herpesviridae family, a significant human pathogen (1). The prevalence of antibody indicating previous infection increases with age in different populations that have been studied. The prevalence of past exposure to CMV, as indicated by a positive IgG, varies markedly throughout the different areas of world and is close to 100% in adults in many developing countries such as the Philippines and Uganda. In the developed countries the prevalence is lower and is approximately 40% at age 20 and 80% at age 60 (2). After primary infection, CMV persists as a sub-clinical, lifelong latent infection in bone marrow–derived myeloid lineage cells (3). 

**Table 1:** Primers used for UL97 gene amplification and sequencing

**Target gene **	**Function**	**Name**	**Sequence (5´ 3´)**
UL97	First-round PCR (outer primers)	UL97PCR1S UL97PCR1AS	F: GTGCTCACGGTCTGGATGT R: CGGTGGGTTTGTACCTTCTC
UL97	Second-round PCR (inner primers) and sequence reaction	UL97PCR2S UL97PCR2AS	F: GCACAACGTCACGGTACATC R: ACCTTCTCTGTTGCCTTTCC

**Table 2:** Patient’s characteristics, viral loads, and CMV UL97 phosphotransferase mutations

**Patient**	**Sex**	**Gene**	**Sequencing**	**Viral Load(copies/mL)**	**Resistance mutation**	**Novel mutations**	**Ganciclovir exposure (days)** a	**outcome**
1	F	UL97	A594V	9×10^4^	YES	NO	95	Allograft loss
2	M	UL97	M460T	4.6×10^4^	YES	NO	184	Allograft loss
3	M	UL97	H520Q	8×10^6^	YES	NO	166	Allograft loss
4	F	UL97	D605E	4 × 10^3^	NO	NO	0	No relapse
5	M	UL97	D605E	3.6 × 10^3^	NO	NO	0	No relapse
6	M	UL97	D605E	4.2 × 10^4^	NO	NO	0	No relapse

a Cumulative duration of induction and intravenous or oral maintenance treatments.

In immunocompetent individuals, primary infections are mostly subclinical and self-limited. In contrast, infections in immunocompromised persons including organ transplant recipients are accompanied by some important morbidity and mortality (4). CMV remains the most common infection affecting organ transplant recipients. Strategies to prevent CMV have significantly reduced CMV disease and decreased the effects of CMV infection (5, 6). Since first use of ganciclovir (GCV) in prevention and treatment regimens according to the recommendations of different consensus documents, Morbidity and mortality of CMV infection in solid organ transplantation has been decreased (7).

It was in the 1980s that Resistance of CMV to antiviral agents was first observed in the laboratory(8) and subsequently these resistant isolates were detected in immunocompromised hosts (9). Resistance of CMV to antiviral agents arises mainly from Mutation within the UL97 and UL54 genes. The UL97, is a gene encoding for protein kinase, while the UL54 encoding for DNA polymerase (10). Occurrence of resistance mutations without prior antiviral drug exposure is very rare and most commonly occurs after prolonged treatment with CMV antiviral therapy (5, 11).

Among The 3 common antiviral drugs, ie, GCV, foscarnet, and cidofovir, ganciclovir targets both UL97 and the UL54 gene where foscarnet and cidofovir target just UL54 gene (12, 13). In about 90 percent cases that are resistant to GCV, mutations in the UL97 phosphotransferase gene are the reason, followed later by the addition of UL54 mutations that confer increased GCV resistance (5, 10, 14). Mutations in UL97 gene don’t induce resistance to foscarnet and cidofovir but UL54 drug resistance mutations occur in the conserved functional domains and can affect susceptibility to foscarnet and cidofovir (5).

Since GCV is the first-line treatment for CMV infections in immunocompromised patients (5), and Common mechanisms of CMV resistance to GCV have been described chiefly with the UL97 mutations, we focused on UL97 mutations and its association with resistance to GCV in this study.

*Patients and methods*

*Patient population and CMV isolates*

The aim of this study was to investigate prevalence of GCV resistance in CMV-positive renal transplant recipients and its association with UL97 gene mutations. We utilized clinical specimens submitted to one of the private Virology Laboratory in Tehran for CMV quantitative measurement. A total of 144 post kidney transplant patients (from different transplant centers in Tehran), with quantitative PCR > 2000 copies/mL were included in the study for UL97 sequencing. After obtaining whole blood from patients’ plasma viral DNA was extracted using QIAamp DNA Minikit (Qiagen), in accordance with the manufacturer’s instructions. Viral DNA purification efficiency was analyzed by means of a dual-color detection system supplied with the kit and then they were stored at -20 ^0^C until tested. In accordance with Shahid Beheshti University of Medical Sciences law, only patients who consented to the review of their medical records were included.

*UL97 gene analysis*

Drug resistance was evaluated for the UL97 region only by a readily available assay using detection methods previously reported (15), Briefly, The extracted viral genomic DNA were amplified in nested PCRs to produce sequencing templates for dideoxy sequencing (BigDye v.3.1, AB) of UL97 kinase. 2 primer sets were used, targeting the 912-bp region of UL97 (from codons 405 to 708), (table 1). Sequencing was done by using the inner primers. Sequences were compared with the AD169 reference sequence for variants affecting amino acid encoding.

## Results

*Patients and virus isolates*

One hundred and forty-four CMV isolates (all recovered from blood), from 144 immunocompromised patients (post kidney transplantation) with viral load >2000 copies/mL (with a range of 2.9 × 10^3^ to 8×10^6^ copies/mL serum) were studied. 

112 patients had received treatment with GCV and 32 patients had not received any anti-CMV drug. None of the patients had been treated with cidofovir or foscarnet. 112 patients who had received treatment with GCV had different duration of GCV administration with median duration of GCV administration of 162 days (range, 13–381 days). Clinical data and results of genotypic studies are summarized in Table 2. 

*Definition of drug resistance and UL97 gene mutation*

Definition of drug resistance: (no improvement (or with relapses) in CMV viremia or clinical disease during prolonged antiviral therapy especially in the presence of risk factors. Generally, prolonged therapy means 6 or more weeks of cumulative antiviral drug exposure, including more than 2 weeks of ongoing full dose therapy at the time of evaluation). (5)

Analysis of CMV DNA by sequencing revealed the presence of 6 single UL97 gene mutations in six patients who had received treatment with GCV (Table 2).

Three patients (2.6%) of 112 patients with history of GCV treatment had clinical resistance with single UL97 mutations at loci known to be related to resistance including an Ala-to-Val mutation at codon 594, MET-to-Thr mutation at codon 460 , and His-to-Gln mutation at codon 520. 3 patients had UL97 mutation without clinical resistance. No clinical resistance or UL97 mutation were observed among patients who were anti-CMV drug naïve. 

The 6 patients with UL97 mutations were followed for 6 month to see the outcomes.

*Ganciclovir resistance and clinical outcome*

Demographic and clinical characteristics and the outcomes of the 3 patients with UL97 confirmed mutations without clinical resistance and 3 patients with UL97 mutations and clinical drug-resistant are presented in Table 2. During 6 months follow-up, three patients with UL97 mutations and clinical resistance had allograft loss. Causes of allograft loss included foscarnet or cidofovir inaccessibility and force to discontinuation of immunosuppression drugs.

In 3 patients with UL97 mutations without clinical resistance no relapses were seen.

## Discussion

Our goal in this study was to determine incidence of ganciclovir resistance in CMV-positive renal transplant recipients and its association with UL97 gene mutations in Iranian post kidney transplant patients. 

In this study, we analyzed viral DNA in the blood of 144 post kidney transplantation patients (32 patients were anti-CMV drug naïve) with CMV (> 2000 copies/mL) in order to determine incidence of GCV resistance and identify mutations in the CMV UL97 gene associated with clinical resistance to GCV. Clinical GCV resistance with UL97 conformed mutations were detected in 3 of 112 patients (2.6%) with history of GCV treatment. No clinical resistance or UL97 mutation were observed among patients who were anti-CMV drug naïve. 

GCV has been the drug of choice for treatment of systemic CMV disease. However, long-term therapy, ongoing active viral replication due to factors such as the lack of prior CMV immunity (D+/R-), high levels of immunosuppressive therapy, and suboptimal drug concentrations increase the risk of development of GCV resistance. The UL97 gene is by far the most frequent site of mutations conferring GCV resistance, and extensive work by a number of groups established that nucleotide changes associated with GCV resistance are clustered at codons 460, 520, and 590 to 607(canonical mutations((12, 16-21).

Ganciclovir resistance reported in 0 to 12 percent in solid organ transplantation with history of GCV treatment in different studies (5, 22) and as mentioned it was 2.6% in our study.

The cumulative data presented here confirm and extend our earlier findings on the specific mutations in the UL97 phosphotransferase gene of clinical CMV isolates resistant to GCV. In 3 of 6 patients with clinically resistant CMV, we found well described UL97 mutations that are known to confer resistance to GCV (patients’ no. 1, 2, and 3). These mutations are A594V, M460T, and H520Q (12, 15). In patients’ no. 4, 5, and 6 we found three common mutations (D605E) with no clinical GCV resistance (Table 2). D605E mutations have controversial role in GCV resistance. Most studies showed that this mutations confer no clinical GCV resistance and may be regarded as a natural sequence variant while some studies have revealed that double mutations A594P/D605E and M460V/D605E have conferred GCV resistance and believe that the high frequency of D605E variation might easily give rise to ganciclovir-resistant CMV in the future (23-25). On the other hand Hosseini et al reported some cases with single D605E mutation and GCV resistance (26, 27). Role of D605E mutations remain controversial and demand further in depth investigations to determine its role in different populations and also its role as a single or double mutation.

The outcome of patients with CMV disease because of ganciclovir-resistant CMV is generally poor, manifested by a high rate of allograft loss as illustrated in our study and those of others (28-30). In our series, all three patients with drug-resistant CMV and no response to GCV, lost their allograft. The treatment of drug-resistant CMV disease is highly complex and often prolonged. No controlled trial data define a best practice for selection of alternate therapy when suspected or confirmed drug resistance is present based on clinical risk factors or genotypic testing. although causes of allograft loss in our study included foscarnet or cidofovir inaccessibility and force to discontinuation of immunosuppression drugs, but modalities that are recommended for the treatment of drug-resistant CMV (e.g. use of foscarnet or cidofovir or reduction of immunosuppression) are also nephrotoxic and can lead to increase in allograft rejection risk and at last allograft dysfunction or even allograft loss (5, 30).

In conclusion, CMV disease caused by drug resistant virus is not an uncommon consequence of widespread and increasing rate of GCV use and increase in the incidence of drug-resistant CMV could be subsequent issue. The observations in this study may therefore foreshadow the emergence of drug-resistant CMV as an entity that could threaten the outcome of transplantation. The morbidity of clinical disease because of drug-resistant CMV, the complexity of its treatment, and the poor outcome of patients should be taken into consideration in the determination of concise role of different mutations and their association with clinical resistance and afterword development of optimized strategies for prevention of drug-resistant CMV and also development of drugs with less toxicity.
